# miR-146a Attenuates Inflammatory Pathways Mediated by TLR4/NF-*κ*B and TNF*α* to Protect Primary Human Retinal Microvascular Endothelial Cells Grown in High Glucose

**DOI:** 10.1155/2016/3958453

**Published:** 2016-02-21

**Authors:** Eun-Ah Ye, Jena J. Steinle

**Affiliations:** ^1^Department of Anatomy and Cell Biology, Wayne State University, Detroit, MI 48201, USA; ^2^Department of Ophthalmology, Wayne State University, Detroit, MI 48201, USA

## Abstract

Pathological mechanisms underlying diabetic retinopathy are still not completely understood. Increased understanding of potential cellular pathways responsive to hyperglycemia is essential to develop novel therapeutic strategies for diabetic retinopathy. A growing body of evidence shows that microRNA (miRNA) play important roles in pathological mechanisms involved in diabetic retinopathy, as well as possessing potential as novel therapeutic targets. The hypothesis of this study was that miR-146a plays a key role in attenuating hyperglycemia-induced inflammatory pathways through reduced TLR4/NF-*κ*B and TNF*α* signaling in primary human retinal microvascular endothelial cells (REC). We cultured human REC in normal (5 mM) glucose or transferred to high glucose medium (25 mM) for 3 days. Transfection was performed on REC with miRNA mimic (hsa-miR-146a-5p). Our results demonstrate that miR-146a expression was decreased in human REC cultured in high glucose. Overexpression of miR-146a using mimics reduced the levels of TLR4/NF-*κ*B and TNF*α* in REC cultured in high glucose. Both MyD88-dependent and -independent signaling were decreased by miR-146a overexpression in REC in high glucose conditions. The results suggest that miR-146a is a potential therapeutic target for reducing inflammation in REC through inhibition of TLR4/NF-*κ*B and TNF*α*. Our study will contribute to understanding of diabetic retinal pathology, as well as providing important clues to develop therapeutics for clinical applications.

## 1. Introduction

Proinflammatory factors are potential mediators associated with pathological mechanisms of diabetic complications, and they can lead to the alteration of epigenetic mechanisms such as microRNAs [1]. Hyperglycemia is a significant risk factor that contributes to the onset and progression of diabetic retinopathy [2–4], and our lab and others have investigated and revealed molecular and cellular mechanisms underlying the pathology of hyperglycemia and diabetic retinopathy [5–9]. However, the mechanisms are still poorly understood and extensive further studies are required to develop novel therapeutic strategies for diabetic retinopathy.

MicroRNA (miR), small noncoding RNA molecules of 21 to 23 nucleotides, are an emerging key regulator of gene expression. miR possess a great potency as a potential biomarker for diagnosing diseases [10–12] and as a regulator of a variety of biological activities, including cellular proliferation, differentiation, development, and death [13]. Only a small number of studies on microRNAs, including miR-15b, -16, -146a/b, -195, and -200b, have been done to investigate the role of miR in diabetic retinopathy and/or hyperglycemia [6, 14–17]. There are still many unanswered questions on the functions of these miRNA, as well as how specific miRNA(s) may affect the pathological mechanisms common in diabetic retinopathy.

miR-146a has been implicated to function in inflammation, innate immunity, and cancer, as well as shown to regulate mitochondrial functions involving inflammation-aging [18–20]. A previous study showed miR-146a expression in different types of retinal cells, including retinal endothelial cells, Müller cells, and RPE cells [21]. Decreased levels of miR-146a expression were reported in peripheral blood mononuclear cells (PBMCs) from type 2 diabetes (T2D) patients [22], and modified rhythmicity of miR-146a expression was shown in REC from diabetic donors [21]. In addition, a negative correlation of miR-146a levels and inflammatory and ER stress markers was reported in patients with T2D [23].

High motility group box 1 (HMGB1) responds to pathogens, both exogenous and endogenous, to activate TLR4, leading to phosphorylation of NF-*κ*B and increased production of proinflammatory molecules in endothelial cells [24]. In addition, the levels NF-*κ*B and TNF*α*, which are downstream mediators of the TLR4 pathway, were reported to be upregulated in the retina of type 2 diabetic rats [25].

Toll-like receptors (TLRs) play an essential role in inflammatory responses, and upregulated levels of TLR4/NF-*κ*B signaling were shown in human microvascular endothelial cells cultured in high glucose conditions [26]. TLR4 levels were upregulated to induce proinflammatory cytokines, activating both MyD88-dependent and -independent pathways, in the retina of streptozotocin-induced diabetic rats [27].

miR-146a regulates TLR-mediated NF-*κ*B activation through a negative feedback loop in human monocytes [28]. NF-*κ*B also plays a role in inflammatory responses and its increased activity is one of the key mechanisms underlying diabetic retinopathy [17, 29–31]. Also, activation of NF-*κ*B is induced by hyperglycemia in REC [31, 32]. Regulatory networks between miR-146a and NF-*κ*B have been reported in HUVEC and brain endothelial cells [33, 34].

It is important to uncover cell type-specific mechanisms of proinflammatory pathways and the interaction of these pathways with miR-146a as a potential therapeutic for diabetic retinopathy. In the present study, we tested the hypothesis that overexpression of miR-146a protects REC from hyperglycemia-induced proinflammatory responses through downregulation of TLR4, NF-*κ*B, and TNF*α*.

## 2. Materials and Methods

### 2.1. Cell Culture

Human REC were acquired from Cell Systems Corporation (CSC, Kirkland, WA). Cells were grown in M131 medium containing microvascular growth supplement (Invitrogen), 10 *μ*g/mL gentamycin, and 0.25 *μ*g/mL amphotericin B. For experiments, cells were maintained in normal (5 mM) glucose or transferred to high glucose medium (25 mM) (Cell Systems) for 3 days. Only primary cells within passage 5 were used. Cells were quiesced by incubating in high or normal glucose medium without growth supplementation for 20 hours and used to perform the experiments.

### 2.2. Cell Transfection with microRNA Mimics

REC were transfected with miRNA mimic (hsa-miR-146a-5p) (Invitrogen, Carlsbad, CA) using Oligofectamine (Invitrogen) following the manufacturer's instructions. miR-transfection was performed 48 hours before cell harvest. A final concentration of 50 nM was used. Additionally, a 50 nM Mimic Negative Control (Invitrogen) was transfected into REC cultured in high glucose as a control. Cells cultured in normal glucose (NG) and high glucose (HG) were treated with 0 nM mimic transfected with Oligofectamine. MiRNA overexpression was verified using quantitative reverse transcription-polymerase chain reaction and real-time PCR.

### 2.3. Quantitative Real-Time PCR

Total RNA was isolated and purified using the Trizol method and the purity and quantity of RNA were measured using Synergy HTX multimode reader (BioTek; Winooski, VT). For polyA tailing reverse-transcriptase PCR, 5 *μ*g of total RNA was treated with DNase I for 15 min at room temperature (Promega; Madison, WI) and then added polyA using (polyA) polymerase (NEB; Ipswich, MA) at 37°C for 1 h. The final reaction mixtures were extracted with phenol/chloroform, precipitated with isopropanol, and redissolved in 25 *μ*L diethylpyrocarbonate- (DEPC-) treated water. PolyA-tailed RNA (6 *μ*L) was reverse-transcribed into first-strand cDNA using Superscript II reverse transcriptase (Invitrogen) with the oligo-dT adapter primer 5′GCGAGCACAGAATTAATACGACTCACTATAGGTTTTTTTTTTTTVN3′. For PCR, 1 *μ*L of RT product was diluted three times and used as a template in each reaction. The forward and reverse primers were purchased from OriGene (Rockville, MD). GAPDH sequence (OriGene) was used as the internal control. The SYBR-Green-based real-time PCR was performed using the CFX Connect PCR system (BioRad; Hercules, CA). The relative expression of miRNA was calculated based on the formula 2^(−ΔΔCt)^.   ΔΔCt values are ΔCt_exp._ − ΔCt_cont._.

### 2.4. Western Blot Analysis

After rinsing with cold PBS, REC were collected in lysis buffer containing protease and phosphatase inhibitors and scraped into tubes. Equal amounts of protein were separated on precast tris-glycine gels (Invitrogen, Carlsbad, CA) and then blotted onto a nitrocellulose membrane. After blocking in TBST (10 mM Tris-HCl buffer, pH 8.0, 150 mM NaCl, and 0.1% Tween 20) and 5% (w/v) BSA, the membrane was treated with appropriate primary antibodies followed by incubation with secondary antibodies labeled with horseradish peroxidase. Antigen-antibody complexes were detected by chemiluminescence reagent kit (Thermo Scientific, Pittsburgh, PA). Primary antibodies used were HMGB1, TLR4, MyD88, TRAF6, IRAK1, IRF3, TRIF, phosphorylated NF-*κ*B p65 (Ser 536), and NF-*κ*B p65 (all purchased from Cell Signaling, Danvers, MA) and beta actin (Santa Cruz, Santa Cruz, CA).

### 2.5. ELISA Analysis

TNF*α* protein concentrations were measured using a TNF*α* ELISA (ThermoFisher, Pittsburgh, PA). 100 *μ*g protein was loaded into all wells, with analyses based on a standard curve. The manufacturer's instructions were followed.

### 2.6. Statistics

Statistical analyses were done using Prism software (GraphPad, La Jolla, CA). Analyses were done using unpaired Student's *t*-test. Data are presented as mean ± SEM. For Western blots, a representative blot is presented.

## 3. Results

### 3.1. miR-146a Expression Was Reduced in Hyperglycemia in REC

Previous work showed miR-146a expression in different types of retinal cells, including retinal endothelial cells, Müller cells, and RPE cells. REC showed relatively low levels of expression of miR-146a, compared to Müller cells [21]. Also, decreased levels of miR-146a expression in peripheral blood mononuclear cells (PBMCs) from T2D patients [22] and modified rhythmicity of miR-146a expression in REC from diabetic donors [21] have been reported.

**Figure 1:**
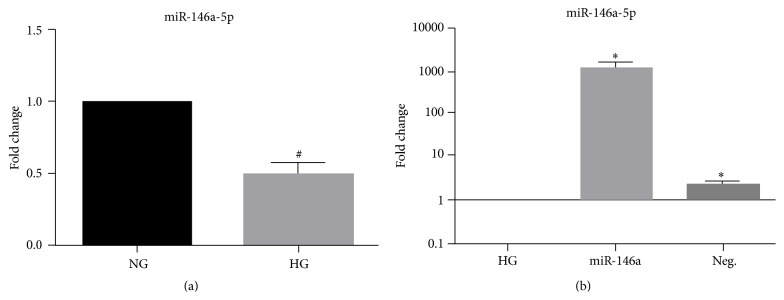
Decrease in miR-146a expression in REC in high glucose and transfection-induced fold changes. (a) Fold changes of miR-146a expression are shown. After 3 days of REC culture in high glucose (25 mM) medium, the expression of miR-146a was reduced, 0.5-fold change, compared to that of normal glucose (NG; 5 mM) group. (b) Transfection-induced fold changes of miR-146a expression in REC. REC were transfected with mimics (50 nM of final concentration) of miR-146a to increase the level of expression in a hyperglycemic condition. The *y*-axis is a logarithmic scale. ^#^ *p* < 0.05 versus NG, ^*∗*^ *p* < 0.05 versus HG; *N* = 4. Data are mean ± SEM.

We measured miR-146a expression in REC after exposure to high glucose. REC were cultured in normal or high glucose medium (25 mM) and total RNA was isolated from the cells, followed by quantitative real-time PCR. High glucose reduced the expression of miR-146a, as compared to normal glucose group (Figure 1(a)). The results indicate that hyperglycemia decreased the level of miR-146a expression in REC.

Since hyperglycemia resulted in decreased expression of miR-146a, we wanted to increase miRNA expression through transfection with miRNA mimics. REC were transfected with miR-146a mimic at a final concentration of 50 nM for 48 hours. Significant increases of miR-146a fold change were confirmed by quantitative real-time PCR (Figure 1(b)). Negative control for the mimic showed limited miR-146a expression in REC cultured in high glucose.

### 3.2. miR-146a Decreased the Levels of HMGB1 in Hyperglycemia

**Figure 2:**
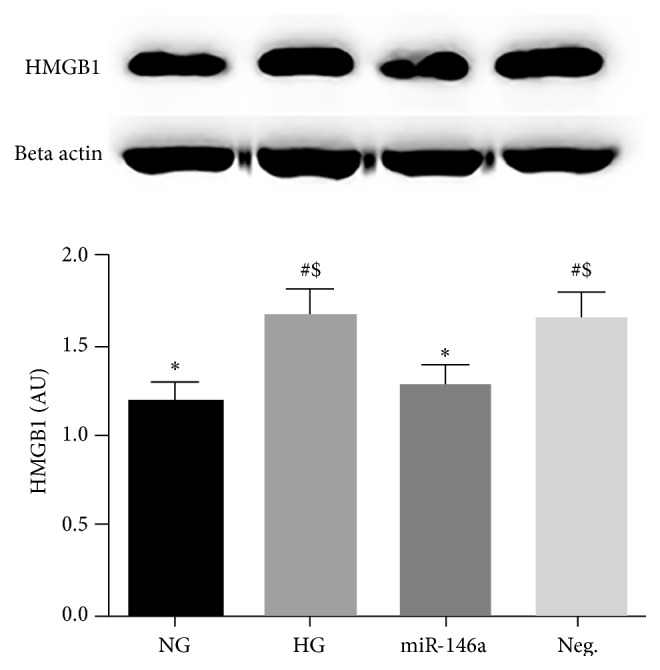
Effects of miR-146a on HMGB1 levels in hyperglycemia. REC were cultured in normal glucose (5 mM, NG) or high glucose (25 mM, HG). High glucose increased HMGB1 levels, which were significantly decreased in REC after miR-146a overexpression. A representative blot is shown. ^#^ *p* < 0.05 versus NG, ^*∗*^ *p* < 0.05 versus HG, and ^$^ *p* < 0.05 versus miR-146a; *N* = 3. Data are mean ± SEM.

HMGB1 signals to TLR4 and plays an important role in mediating proinflammatory responses [24]. The levels of HMGB1 signaling were increased in the retina with T2D and ARPE-19 cells treated with high glucose [25]. A study showed potential association of miR-146a with HMGB1 in a peritonitis model [35]. We are unaware of any studies on the miRNA regulation of HMGB1 levels in hyperglycemia and diabetic retinopathy. Thus, we examined whether miR-146a plays a role in reducing the levels of HMGB1 in REC in hyperglycemia. We found that the increased levels of HMGB1 found in REC cultured in high glucose were significantly reduced in REC, which overexpressed miR-146a (Figure 2). This suggests that miR-146a may inhibit HMGB1 signaling in REC under high glucose conditions.

### 3.3. miR-146a Reduced TLR4 Signaling in Hyperglycemia

**Figure 3:**
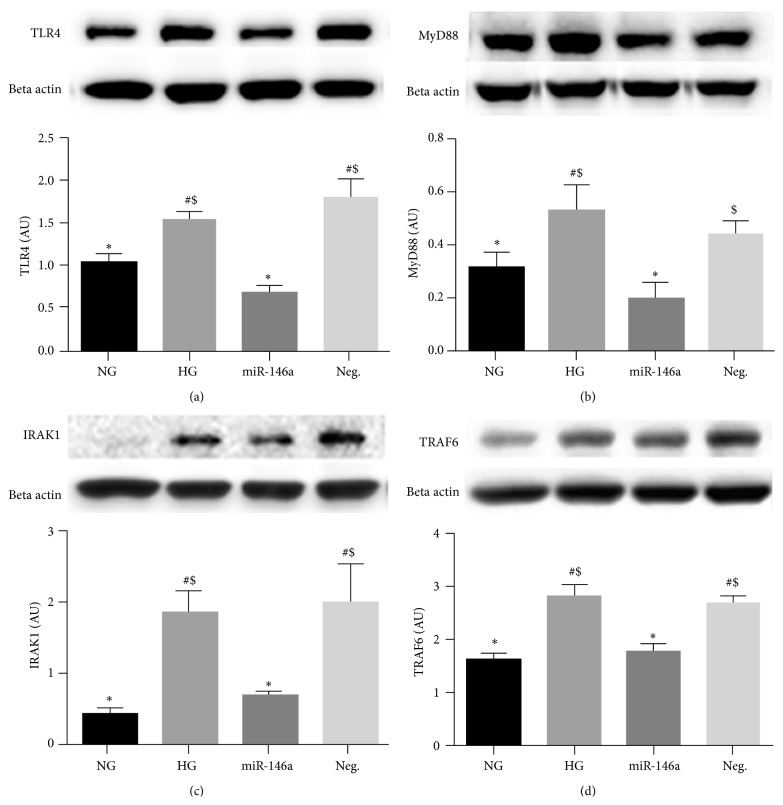
Protein levels of TLR4, MyD88, IRAK1, and TRAF6 levels in REC. REC were cultured in normal glucose (5 mM, NG) or high glucose (25 mM, HG). REC transfected with miR-146a mimic had reduced levels of TLR4, MyD88, IRAK1, and TRAF6, compared to that of control HG group. A representative blot is shown. ^#^ *p* < 0.05 versus NG, ^*∗*^ *p* < 0.05 versus HG, and ^$^ *p* < 0.05 versus miR-146a. A minimum of *N* = 3 was used for all data. Data are mean ± SEM.

It has been reported that high glucose culturing conditions can elevate TLR4 protein levels in REC. That, in turn, increased both MyD88-dependent and -independent (IRF3 and TRIF) signaling [36]. TRAF6 and IRAK1, downstream molecules of MyD88-dependent signaling, have been reported as direct targets of miR-146a [37, 38]. Our results demonstrated that overexpression of miR-146a in REC resulted in decreased levels of TLR4 and MyD88 in REC cultured in high glucose compared to untransfected cells (Figures 3(a) and 3(b)). In addition, signaling pathway members downstream of the MyD88-dependent pathway, IRAK1 and TRAF6, were also decreased in REC when miR-146a was overexpressed (Figures 3(c) and 3(d)).

**Figure 4:**
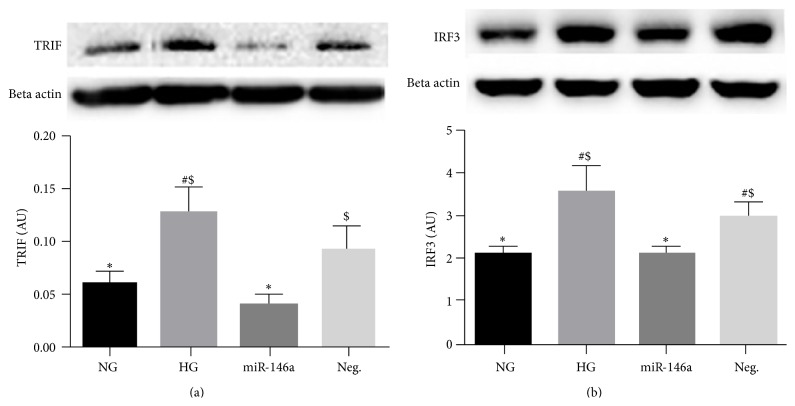
Changes of MyD88-independent signaling, TRIF and IRF3 levels, in REC. REC were cultured in normal glucose (5 mM, NG) or high glucose (25 mM, HG). Overexpression of miR-146a decreased the levels of TRIF (a) and IRF3 (b) in REC, compared to that of control HG group. A representative blot is shown. ^#^ *p* < 0.05 versus NG, ^*∗*^ *p* < 0.05 versus HG, and ^$^ *p* < 0.05 versus miR-146a. A minimum of *N* = 3 was used for all data. Data are mean ± SEM.

We also demonstrated that members of a MyD88-independent pathway, TRIF and IRF3, were also decreased by miR-146a overexpression in REC cultured in high glucose (Figures 4(a) and 4(b)). These results indicate that miR-146a plays a role in reducing proinflammatory signaling through the inhibition of TLR4 and its downstream signaling pathways.

### 3.4. miR-146a Decreased the Levels of NF-*κ*B in Hyperglycemia

**Figure 5:**
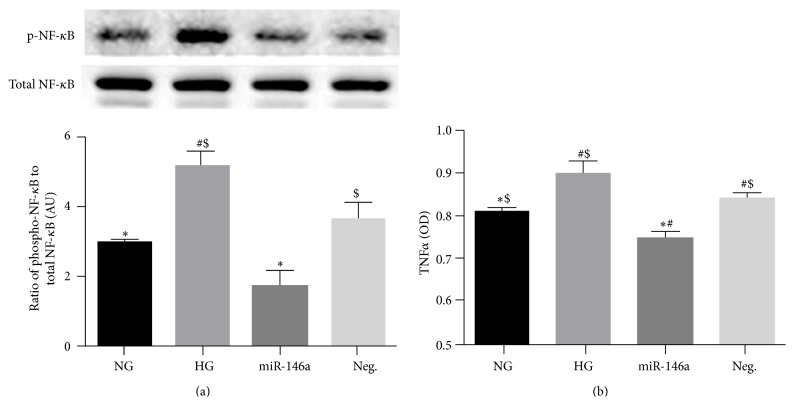
Effects of miR-146a on NF-*κ*B (Ser 536) phosphorylation and TNF*α* levels in hyperglycemia. REC were cultured in normal glucose (5 mM, NG) or high glucose (25 mM, HG). (a) Overexpression of miR-146a decreased the levels of NF-*κ*B phosphorylation, which was elevated in control HG condition. A representative blot is shown. (b) ELISA data for TNF*α* on REC in normal glucose (NG, 5 mM) or high glucose (HG, 25 mM) and transfected groups. miR-146a decreased TNF*α* levels significantly, compared to control HG condition. ^#^ *p* < 0.05 versus NG, ^*∗*^ *p* < 0.05 versus HG, and ^$^ *p* < 0.05 versus miR-146a; *N* = 3. Data are mean ± SEM.

Activation of NF-*κ*B is induced in hyperglycemia in REC [31, 32], which can be induced by increased TLR4 levels under high glucose conditions [36]. Therefore, we questioned whether miR-146a affected NF-*κ*B phosphorylation in REC under high glucose conditions. We found that high glucose treatment increased NF-*κ*B p65 phosphorylation, with miR-146a transfection significantly inhibiting this phosphorylation compared to nontransfected REC cultured in high glucose conditions (Figure 5(a)). The results indicate that miR-146a inhibits NF-*κ*B activation in response to high glucose to protect REC.

### 3.5. miR-146a Decreased the Levels of TNF*α* in Hyperglycemia

We previously reported that TNF*α* levels are increased in REC under high glucose conditions [7]. In addition, TNF*α* levels can be elevated by the activation of TLR4 signaling [39]. In this study, we found that miR-146a overexpression significantly reduced TNF*α* levels in REC cultured in high glucose (Figure 5(b)). Therefore, data suggests that high glucose-induced elevation of TNF*α* was decreased in REC when miR-146a was overexpressed.

## 4. Discussions

A growing body of scientific evidence has suggested a regulatory role and the potential impact of microRNAs in treatment for diabetic retinopathy. miR-146a has been reported as one of the potential epigenetic regulators, affecting cellular pathways underlying inflammatory responses in various cell types [34, 40–43]. Our previous work and other studies have demonstrated that REC are a crucial cell type substantially affected in diabetic retinopathy [44–46]. However, the expression and function of miRNA is cell type- and tissue-specific. The regulation of proinflammatory pathways by miR-146a on human REC was unclear. In the present study, we aimed to investigate changes in miR-146a regulation of proinflammatory signaling in REC cultured under high glucose conditions.

MiR-146a expression was reported in human and bovine REC [21, 47]. In this study, we showed that miR-146a expression was decreased in human REC under high glucose conditions, which agrees well with other studies showing a downregulation of miR-146a in HUVEC cells cultured under high glucose conditions [47]. In contrast, miR-146a expression was increased in human renal glomerular endothelial cells cultured in high glucose [48] and in REC of STZ-induced diabetic rats compared to control rats [49]. The differences in miR-146a expression may be due to cell type-specific and specific condition-dependent responses of miRNA.

Little information exists on the relation of HMGB1 and miRNA in the cellular mechanisms of diabetic retinopathy. In the present study, we demonstrated that overexpression of miR-146a induced the decrease of HMGB1 levels in REC in hyperglycemia. HMGB1 plays a crucial role in mediating inflammatory responses [50]. HMGB1 is expressed in endothelial cells, and HMGB1-signaling can induce NF-*κ*B [24]. Previous studies have shown a correlation of HMGB1 to diabetic retinopathy. The levels of serum HMGB1 were upregulated in the patients with T2DM which was correlated with serum TNF*α* [51], and direct effects of HMGB1 on the death of retinal endothelial cells were shown in an animal model of neovascularization [52]. Another study showed that the levels of HMGB1 were decreased in peritoneal lavage fluid supernatants, accompanied by reduced expression of miR-146a in peritoneal exudate cells of LPS-treated mice [35].

TLR4 signaling is one of the downstream pathways activated by HMGB1, playing a significant role in the pathogenesis of inflammation [50]. A negative correlation was shown between miR-146a and MyD88 signaling in epithelial ovarian cancer cells [53]. In lung endothelial cells, the inhibition of TRIF signaling decreased apoptosis and permeability changes after exposure to LPS (an activator of TLR4), while MyD88 inhibition showed no such effects [54]. In human nasal epithelial cells, miR-146a regulated the maintenance of tight junction barrier [55]. However, little has been done to investigate miR-146a and MyD88 in retinopathy-like conditions. Our study demonstrated that both MyD88-dependent and -independent signaling were suppressed by miR-146a overexpression in REC cultured in high glucose. These findings suggest that miR-146a plays a role in reducing proinflammatory signaling via MyD88-dependent and -independent pathways in REC. Further studies on the association of miR-146a with TLR4 and MyD88 pathways will broaden our understanding on the regulation of retinal endothelial permeability and contribute to developing novel therapeutic strategies for the complications of diabetic retinopathy.

A regulatory loop between miR-146a and NF-*κ*B has been reported in a few cell types, including breast cancer cells [56], human monocytes [28], and HUVECs [33]. Our results demonstrated that elevated levels of NF-*κ*B phosphorylation in high glucose were reduced by miR-146a overexpression in REC. This indicates that miR-146a negatively regulated the activity of NF-*κ*B in REC under high glucose conditions. Our results agree with other studies demonstrating that miR-146a inhibited NF-*κ*B signaling, thereby suppressing leukocyte adhesion during neuroinflammation in retinal endothelial cells [17]. Lastly, we demonstrated that miR-146a overexpression resulted in the decrease of hyperglycemia-induced elevation of TNF*α* in REC. Different mechanisms that mediate the elevation of TNF*α* levels have been revealed. High glucose-induced elevation of TNF*α* levels was shown in REC in our previous study [7], and the addition of HMGB1 induced an increase of TNF*α* level in cultured astroglia [57]. Additionally, TNF*α* levels can be elevated by the activation of TLR4 and MyD88-dependent signaling, as shown in cerebral vascular endothelial cells [39]. Our results suggest that miR-146a decreases the levels of hyperglycemia-induced TNF*α* possibly through the inhibition of HMGB1, TLR4, MyD88, and TRIF/IRF3 signaling.

## 5. Conclusions

Taken together, our study demonstrated that high glucose resulted in the reduction of miR-146a expression in REC. miR-146a overexpression suppressed the downstream signaling of TLR4/NF-*κ*B pathway and TNF*α* in REC under high glucose conditions. Therefore, we present a potential regulatory mechanism whereby miR-146a can downregulate TLR4/NF-*κ*B and TNF*α* pathways in REC cultured under high glucose conditions. The outcome suggests that miR-146a is a potential therapeutic target for rescuing diabetic retina through the inhibition of proinflammatory pathways of TLR4/NF-*κ*B and TNF*α*.

## Abbreviations

miR: microRNA
REC: Retinal endothelial cells
PCR: Polymerase chain reaction
ELISA: Enzyme-linked immunosorbent assay
TLR4: Toll-like receptor 4
MyD88: Myeloid differentiation primary response 88
IRAK1: Interleukin-1 receptor-associated kinase 1
TRAF6: TNF receptor-associated factor 6
TRIF: TIR-domain-containing adapter-inducing interferon-*β*
IRF3: Interferon regulatory factor 3
NF-*κ*B: Nuclear factor-*κ*B
TNF*α*: Tumor necrosis factor alpha.
